# Case Report: Contrasting phenotypes of arrhythmogenic cardiomyopathy: classic desmosomal ARVC and a RIT1-related phenocopy

**DOI:** 10.3389/fcvm.2026.1786847

**Published:** 2026-03-16

**Authors:** Bo Eun Park, Dong Heon Yang

**Affiliations:** 1 Division of Cardiology, Department of Internal Medicine, Kyungpook National University Hospital, Daegu, Republic of Korea; 2 Division of Cardiology, Department of Internal Medicine, School of Medicine, Kyungpook National University, Daegu, Republic of Korea

**Keywords:** arrhythmogenic right ventricular cardiomyopathy, cardiomyopathy, desmosomal mutation, phenocopy, RIT1

## Abstract

**Background:**

Arrhythmogenic right ventricular cardiomyopathy (ARVC) is most commonly associated with pathogenic variants in desmosomal genes. However, ARVC-like phenotypes may also arise from non-desmosomal genetic backgrounds, creating diagnostic challenges and raising the concept of phenocopies.

**Case summary:**

We describe two contrasting cases of arrhythmogenic cardiomyopathy. Case 1 is a middle-aged man presenting with atrial fibrillation and imaging evidence of right ventricular (RV) fatty infiltration, repolarization abnormalities, and positive signal-averaged electrocardiogram (ECG)**,** in whom a pathogenic RIT1 variant was identified without desmosomal mutations. This case was interpreted as a RASopathy-associated arrhythmogenic cardiomyopathy phenocopy. Case 2 is a woman with severe RV dysfunction, ventricular arrhythmia burden, characteristic ECG findings, a strong family history of sudden cardiac death, and a likely pathogenic DSG2 variant, fulfilling multiple major Task Force Criteria for classic ARVC and requiring implantable cardioverter-defibrillator implantation.

**Discussion:**

These cases highlight the genetic and phenotypic heterogeneity of ARVC and emphasize the importance of multimodality imaging and extended genetic testing to distinguish classic desmosomal disease from phenocopies.

## Introduction

Arrhythmogenic right ventricular cardiomyopathy (ARVC) is an inherited cardiomyopathy characterized by progressive fibrofatty replacement of the right ventricular myocardium, ventricular arrhythmias, and an increased risk of sudden cardiac death (1). Pathogenic variants in desmosomal genes, including PKP2, DSG2, DSP, and DSC2, account for the majority of genetically confirmed cases. Accordingly, the revised 2010 Task Force Criteria emphasize desmosomal genetics as a major diagnostic component (2).

With the widespread adoption of next-generation sequencing, increasing genetic heterogeneity has been recognized, and the disease concept has expanded from isolated ARVC to the broader spectrum of arrhythmogenic cardiomyopathy, supporting a precision medicine framework that integrates genotype, phenotype, and clinical context (3, 4). ARVC-like phenotypes associated with non-desmosomal genetic variants have increasingly been recognized, giving rise to the concept of phenocopies that may overlap phenotypically with classic desmosomal disease but differ in underlying pathophysiology and clinical implications.

Herein, we present two contrasting cases: one representing classic desmosomal ARVC with severe right ventricular dysfunction and high arrhythmic risk, and the other illustrating an ARVC-like phenotype associated with a pathogenic RIT1 variant, a gene primarily linked to RASopathies. These cases underscore the spectrum of arrhythmogenic cardiomyopathy and the importance of an integrated diagnostic approach.

## Case presentation

### Case 1: RIT1-related arrhythmogenic cardiomyopathy phenocopy

#### Patient information and presenting concerns

A middle-aged man with no previously diagnosed structural heart disease presented to the emergency department with a 2-day history of palpitations, generalized weakness, and dizziness. Electrocardiography on admission revealed atrial fibrillation with a rapid ventricular response.

#### Medical, family, and genetic history

The patient had no known history of cardiomyopathy or prior ventricular arrhythmias. Family history was notable for sudden cardiac death in a maternal uncle (a second-degree relative) in his 50s.

Genetic testing identified a heterozygous RIT1 c.244T>G (p.Phe82Val) variant, which was classified as pathogenic according to the American College of Medical Genetics and Genomics (ACMG) criteria. No pathogenic or likely pathogenic variants in desmosomal genes were detected.

#### Diagnostic assessment

Diagnostic assessment in Case 1 was challenging because the patient demonstrated several arrhythmogenic features without fulfilling contemporary diagnostic criteria for classic ARVC. A multimodality evaluation was therefore undertaken to integrate electrical, structural, and genetic findings.

**Figure 1:**
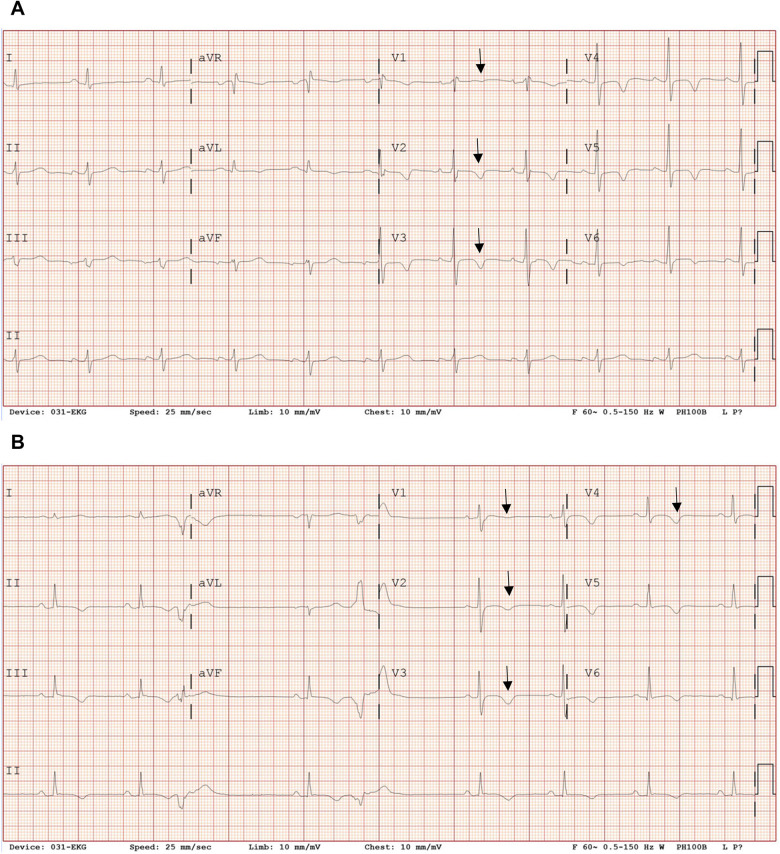
Twelve-lead electrocardiographic findings. Representative resting 12-lead electrocardiograms obtained during sinus rhythm are shown. **(A)** In Case 1, the electrocardiogram demonstrates sinus rhythm with incomplete right bundle branch block and T-wave inversion in the right precordial leads (V1–V3), which may fulfill minor repolarization criteria according to the 2010 Task Force Criteria; however, the repolarization abnormality is interpreted cautiously, as it may represent a secondary change related to the conduction delay. **(B)** In Case 2, the electrocardiogram shows sinus rhythm with T-wave inversion extending across the right precordial leads, consistent with repolarization abnormalities that fulfill diagnostic criteria for classic arrhythmogenic right ventricular cardiomyopathy.

**Figure 2:**
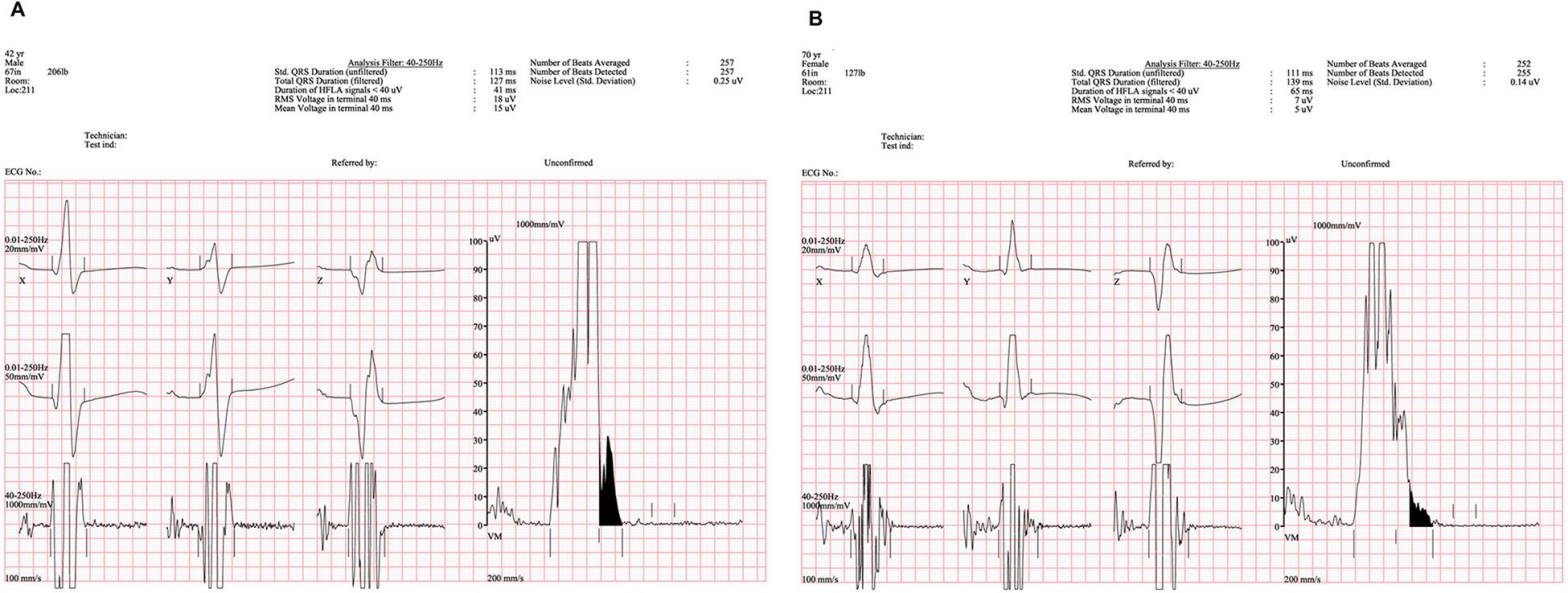
Signal-averaged electrocardiograms demonstrating depolarization abnormalities in both patients. **(A)** In Case 1, signal-averaged electrocardiography shows positive late potentials, with a prolonged filtered QRS duration (127 ms), increased low-amplitude signal duration (<40 μV, 41 ms), and reduced root mean square (RMS) voltage (18 μV). **(B)** In Case 2, signal-averaged electrocardiography demonstrates markedly abnormal depolarization parameters, including a prolonged filtered QRS duration (139 ms), substantially increased low-amplitude signal duration (65 ms), and markedly reduced RMS voltage (7 μV), fulfilling all criteria for positive late potentials according to the 2010 Task Force Criteria.

**Figure 3:**
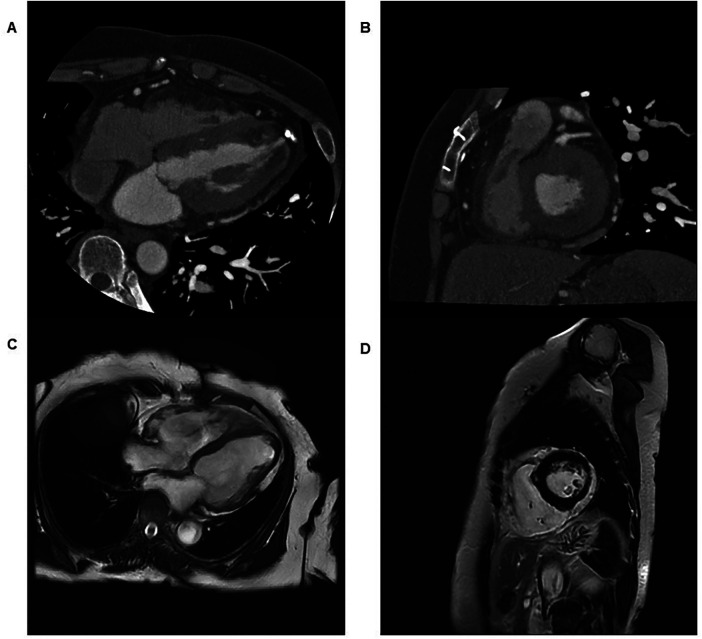
Multimodality imaging findings in two patients with arrhythmogenic cardiomyopathy. **(A)** Axial cardiac computed tomography image from Case 1 demonstrating fatty hypertrophy of the right ventricular free wall. **(B)** Axial cardiac computed tomography image from Case 1 demonstrating subepicardial fatty infiltration of the left ventricular apex, indicating left ventricular involvement. **(C)** Still frame captured from cardiac magnetic resonance cine imaging of Case 2 showing global right ventricular hypokinesia. **(D)** Short-axis cardiac magnetic resonance image from Case 2 demonstrating right ventricular wall thinning and akinesia. (See Supplementary Video S1 for the corresponding cine sequence.).

**Figure 4:**
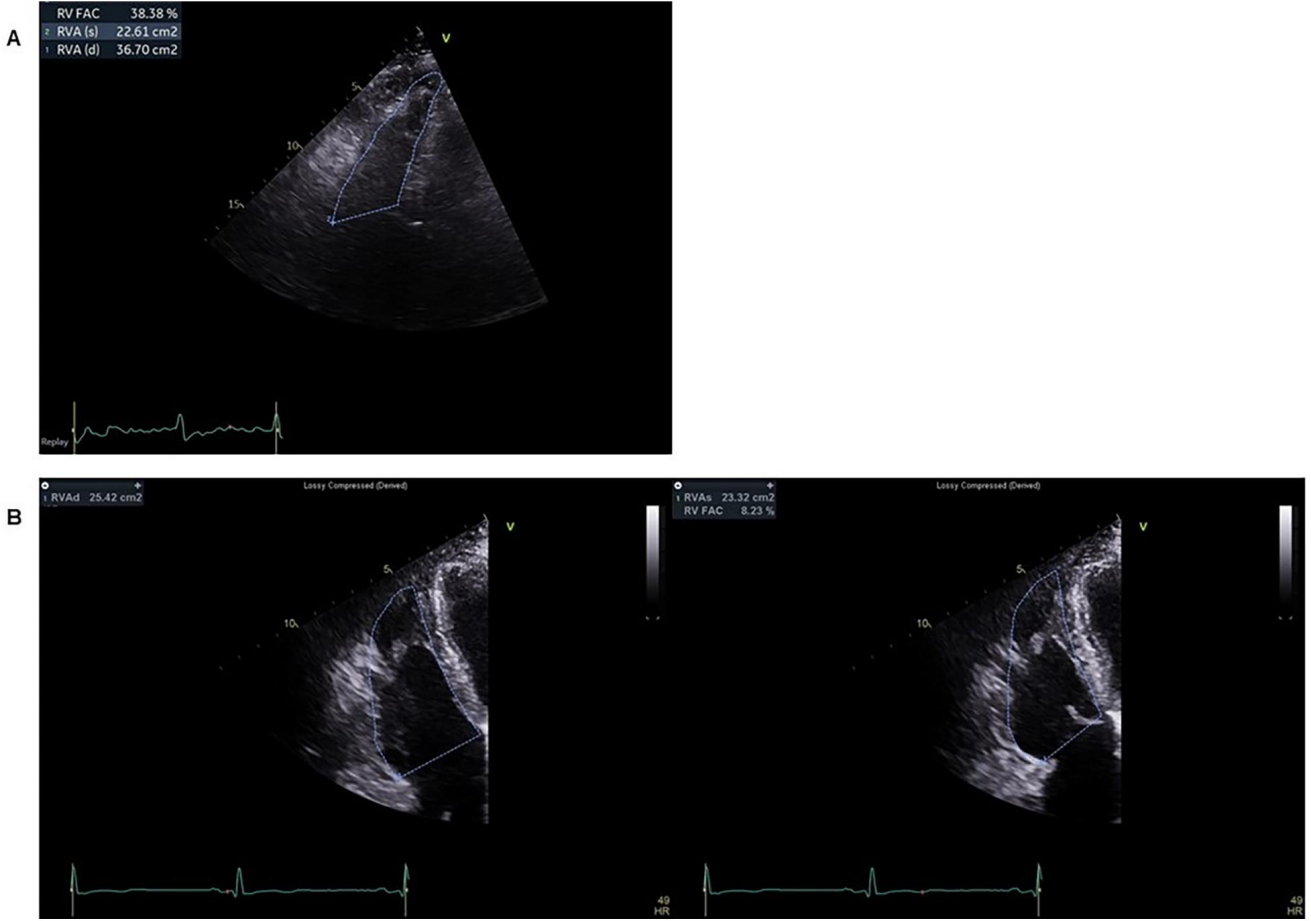
Echocardiographic assessment of right ventricular systolic function. Right ventricular–focused apical four-chamber views are shown to compare right ventricular systolic function between the two cases. **(A)** In Case 1, transthoracic echocardiography demonstrates preserved to borderline right ventricular systolic function, with a right ventricular fractional area change of approximately 38%. **(B)** In Case 2, end-diastolic and end-systolic frames illustrate severe right ventricular systolic dysfunction, with a markedly reduced right ventricular fractional area change of approximately 8%.

Electrocardiography demonstrated incomplete right bundle branch block with T-wave inversion in leads V1–V3 (Figure 1A), fulfilling minor repolarization criteria according to the 2010 Task Force Criteria; however, the repolarization abnormality was interpreted cautiously, as the T-wave inversion may represent a secondary change related to the conduction delay rather than a primary repolarization abnormality. Signal-averaged electrocardiography demonstrated positive late potentials, supporting the presence of an arrhythmogenic substrate (Figure 2A). Coronary computed tomography angiography demonstrated fatty hypertrophy of the right ventricular free wall and focal subepicardial fatty infiltration of the left ventricular apex (Figures 3A,B). Transthoracic echocardiography revealed preserved left ventricular systolic function and preserved to borderline right ventricular systolic function (Figure 4A). Cardiac magnetic resonance imaging could not be performed in Case 1 because of severe claustrophobia, precluding assessment of myocardial fibrosis by late gadolinium enhancement.

Several differential diagnoses were considered, including cardiac sarcoidosis, myocarditis, athlete's heart, and hypertrophic cardiomyopathy associated with RASopathies. Although RIT1 variants are classically associated with hypertrophic cardiomyopathy, there was no echocardiographic or computed tomographic evidence of left ventricular hypertrophy or hypertrophic remodeling.

Taken together, these findings supported the interpretation of a non-desmosomal arrhythmogenic cardiomyopathy phenocopy rather than classic desmosomal ARVC.

#### Therapeutic interventions and follow-up

Therapeutic intervention in Case 1 focused on rhythm control for atrial fibrillation and conservative management of arrhythmic risk. Atrial fibrillation with rapid ventricular response was treated with direct-current cardioversion.

During follow-up, the patient experienced recurrent atrial fibrillation associated with acute decompensated heart failure, prompting repeat cardioversion and optimization of medical therapy.

Given the absence of documented ventricular arrhythmias, preserved right ventricular systolic function, and incomplete fulfillment of diagnostic criteria for classic ARVC, implantable cardioverter-defibrillator implantation was not pursued.

During follow-up, the patient was regularly evaluated in the outpatient clinic with clinical assessment and rhythm monitoring. Clinician-assessed outcomes demonstrated stable functional status without progression of heart failure symptoms between episodes of atrial fibrillation.

Ambulatory rhythm monitoring during follow-up revealed no sustained ventricular arrhythmias. Transthoracic echocardiography showed stable left ventricular systolic function and no deterioration in right ventricular systolic function. The patient remained adherent to medical therapy and tolerated treatment without significant adverse effects. No unanticipated adverse events were observed during follow-up.

### Case 2: classic desmosomal arrhythmogenic right ventricular cardiomyopathy

#### Patient information and presenting concerns

A woman presented with paroxysmal supraventricular tachycardia that terminated with adenosine. Subsequent evaluation for heart failure symptoms revealed no coronary artery disease on coronary CT angiography.

#### Medical, family, and genetic history

Family history was notable for sudden cardiac death in two first-degree relatives. The family history was remarkable for sudden cardiac death in the patient's brother at 49 years of age and in the patient's mother at 69 years of age. Genetic testing identified a likely pathogenic DSG2 c.137G>A (p.Arg46Gln) variant and a variant of uncertain significance in DSC2.

#### Diagnostic assessment

Cardiac magnetic resonance imaging demonstrated severe right ventricular (RV) systolic dysfunction (right ventricular ejection fraction of 31%), global RV hypokinesia, and inferior wall thinning with akinesia, consistent with major structural criteria and previously reported prognostic markers in arrhythmogenic right ventricular cardiomyopathy (Figures 3C,D).

Quantitative assessment further demonstrated an indexed right ventricular end-diastolic volume of 93.41 mL/m^2^, supporting significant right ventricular structural and functional abnormalities.

Echocardiography confirmed severe RV dysfunction with a markedly reduced fractional area change of approximately 8%, consistent with advanced disease (Figure 4B). Left ventricular systolic function was initially reduced but recovered to >50% during follow-up.

Electrocardiography revealed deep T-wave inversion in leads V1–V4, consistent with repolarization abnormalities typical of arrhythmogenic right ventricular cardiomyopathy (Figure 1B). Signal-averaged electrocardiography was positive for late potentials, reflecting delayed activation and conduction abnormalities typical of arrhythmogenic right ventricular cardiomyopathy (Figure 2B).

Holter monitoring demonstrated frequent premature ventricular complexes (>2,000 per 24 h), a known marker of increased ventricular arrhythmic risk in arrhythmogenic right ventricular cardiomyopathy. However, detailed electrocardiographic morphology of the premature ventricular complexes was not consistently available, precluding definitive localization of their site of origin.

#### Therapeutic interventions and follow-up

Given the presence of severe right ventricular dysfunction, frequent ventricular ectopy, and a strong family history of sudden cardiac death, the patient fulfilled multiple major Task Force Criteria for arrhythmogenic right ventricular cardiomyopathy and underwent implantable cardioverter-defibrillator implantation for primary prevention.

Following implantable cardioverter-defibrillator implantation, the patient was followed regularly with device interrogation and clinical evaluation. Clinician-assessed outcomes demonstrated stabilization of heart failure symptoms.

Device follow-up revealed no inappropriate shocks. Ambulatory monitoring confirmed persistent ventricular ectopy without sustained ventricular arrhythmias requiring device therapy. Left ventricular systolic function recovered during follow-up, whereas severe right ventricular dysfunction persisted. The patient demonstrated good adherence to medical therapy and tolerated treatment without device-related complications or unexpected adverse events.

The clinical timelines, key diagnostic findings, management strategies, and outcomes of the two cases are summarized in Table 1.

**Table 1:** Clinical timeline and Key diagnostic findings of the two cases.

Time Point	Case 1: RIT1-related ACM Phenocopy	Case 2: Desmosomal ARVC	Key Clinical Implications
Initial presentation	Palpitations, dizziness; atrial fibrillation with rapid ventricular response	Palpitations; supraventricular tachycardia and progressive heart failure symptoms	Different arrhythmic presentations
Family history	Relative with sudden cardiac death in fifth decade	Sudden cardiac death in brother (49y) and mother (69y)	High inherited risk in both cases
Genetic testing	Pathogenic RIT1 variant; no desmosomal mutation	Likely pathogenic DSG2 variant	Non-desmosomal phenocopy vs. classic ARVC
Key imaging findings	CT: RV fatty hypertrophy; preserved RV function; CMR not performed	CMR: severe RV dysfunction, RVEDVi 93.41 mL/m²	Structural severity differs
Diagnostic limitations	CMR not performed due to claustrophobia; LGE unavailable	CMR performed with RV functional and tissue characterization	Impact of imaging availability on diagnostic certainty
Management	Conservative, phenotype-driven follow-up	ICD implantation for primary prevention	Phenotype-driven risk stratification
Follow-up and outcomes	No sustained ventricular arrhythmias; stable RV function on follow-up	Persistent RV dysfunction; no inappropriate ICD shocks	Divergent clinical trajectories

ACM, arrhythmogenic cardiomyopathy; ARVC, arrhythmogenic right ventricular cardiomyopathy; CMR, cardiac magnetic resonance imaging; CT, computed tomography; ICD, implantable cardioverter-defibrillator; LGE, late gadolinium enhancement; RV, right ventricle; RVEDVi, indexed right ventricular end-diastolic volume.

## Discussion

These two cases illustrate the phenotypic and genetic heterogeneity of arrhythmogenic cardiomyopathy. The second case represents classic desmosomal arrhythmogenic right ventricular cardiomyopathy with severe right ventricular dysfunction, malignant arrhythmic substrate, and a clear indication for implantable cardioverter-defibrillator therapy, in line with contemporary risk stratification models (5, 6). In contrast, the first case demonstrates an arrhythmogenic cardiomyopathy–like phenotype associated with a pathogenic RIT1 variant, a gene traditionally linked to RASopathies and hypertrophic cardiomyopathy.

RIT1 encodes a small GTPase involved in the RAS/MAPK signaling pathway. Although cardiomyopathy in RASopathies is most commonly hypertrophic, dysregulated RAS/MAPK signaling may result in heterogeneous myocardial remodeling, potentially giving rise to arrhythmogenic phenotypes beyond the classic hypertrophic spectrum. The presence of right ventricular fatty infiltration, repolarization abnormalities, and positive signal-averaged electrocardiography findings in our patient supports the interpretation of an arrhythmogenic cardiomyopathy phenocopy rather than coincidental findings, particularly in the context of a non-desmosomal genetic background (3, 7, 8). However, the absence of functional or segregation data linking this specific RIT1 variant to arrhythmogenic phenotypes represents an important limitation.

Distinguishing classic desmosomal arrhythmogenic right ventricular cardiomyopathy from phenocopies has important clinical implications, including exclusion of mimicking conditions such as cardiac sarcoidosis or physiological adaptation in athletes (9, 10). In Case 1, cardiac magnetic resonance imaging could not be performed because of severe claustrophobia, limiting comprehensive myocardial tissue characterization and formal assessment of late gadolinium enhancement. This represents an additional diagnostic limitation and underscores the importance of integrating multimodality imaging with electrocardiographic and genetic findings.

Desmosomal arrhythmogenic right ventricular cardiomyopathy carries a well-established risk of ventricular arrhythmias and sudden cardiac death, guiding implantable cardioverter-defibrillator decision-making. In contrast, risk stratification in non-desmosomal arrhythmogenic cardiomyopathy phenocopies remains less clearly defined. In the absence of sustained ventricular arrhythmias and with preserved right ventricular systolic function, management in Case 1 was guided by phenotype rather than genotype. High-intensity exercise restriction was considered and discussed; however, given the limited arrhythmic burden, management focused on individualized risk assessment, shared decision-making, and close longitudinal follow-up, consistent with contemporary precision medicine approaches in inherited cardiomyopathies (4).

A further limitation of this case report is the absence of functional or segregation data linking the identified RIT1 variant to arrhythmogenic phenotypes, which precludes definitive causal inference. In addition, although both cases were associated with clinically significant family histories of sudden cardiac death, cascade genetic screening and formal segregation analysis of first-degree relatives were not systematically performed, limiting confirmation of variant co-segregation with the disease phenotype.

In addition, the absence of cardiac magnetic resonance imaging in Case 1 precluded evaluation of late gadolinium enhancement, which limits differentiation between an arrhythmogenic cardiomyopathy phenocopy and non-pathologic fatty infiltration.

## Conclusion

ARVC should be regarded as a spectrum of arrhythmogenic cardiomyopathies with diverse genetic substrates. While desmosomal mutations define classic ARVC, non-desmosomal variants such as RIT1 may be associated with phenocopies that share overlapping structural and electrical features. Recognition of this heterogeneity through integrated imaging, electrocardiographic analysis, and genetic evaluation is essential for accurate diagnosis (11–16) and underscores the importance of phenotype-driven, individualized clinical management.

## Data Availability

The original contributions presented in the study are included in the article/Supplementary Material, further inquiries can be directed to the corresponding author.
